# Radar-Based, Simultaneous Human Presence Detection and Breathing Rate Estimation

**DOI:** 10.3390/s21103529

**Published:** 2021-05-19

**Authors:** Nir Regev, Dov Wulich

**Affiliations:** School of Electrical and Computer Engineering, Ben-Gurion University of The Negev, Beer-Sheva 8410501, Israel; dov@ee.bgu.ac.il

**Keywords:** micro-Doppler, occupancy detection, presence detection, vital signs, respiration, spectral-estimation

## Abstract

Human presence detection is an application that has a growing need in many industries. Hotel room occupancy is critical for electricity and energy conservation. Industrial factories and plants have the same need to know the occupancy status to regulate electricity, lighting, and energy expenditures. In home security there is an obvious necessity to detect human presence inside the residence. For elderly care and healthcare, the system would like to know if the person is sleeping in the room, sitting on a sofa or conversely, is not present. This paper focuses on the problem of detecting presence using only the minute movements of breathing while at the same time estimating the breathing rate, which is the secondary aim of the paper. We extract the suspected breathing signal, and construct its Fourier series (FS) equivalent. Then we employ a generalized likelihood ratio test (GLRT) on the FS signal to determine if it is a breathing pattern or noise. We will show that calculating the GLRT also yields the maximum likelihood (ML) estimator for the breathing rate. We tested this algorithm on sleeping babies as well as conducted experiments on humans aged 12 to 44 sitting on a chair in front of the radar. The results are reported in the sequel.

## 1. Introduction

The need for remote human presence detection is growing. Home security systems use cameras and passive infra-red (PIR) sensors to determine if a person approaching or is inside the house. Passive infra-red sensors suffers from many false alarm, but more importantly, they cannot sense minute movements like a still, sleeping person and cameras are not suitable for applications in which privacy is needed like monitoring an elderly in the restroom. Radars have become a readily available solution for the consumer, so in this paper we will focus on the usage of a radar to detect the micro-Doppler effect of breathing, and thereby determine if a person is there or not. The problem of presence sensing is basically an decision problem: is there someone in the radar search volume or not? If the person walks or moves inside the room, a simple moving target indicator (MTI) followed by a constant false alarm rate (CFAR) detector can be employed to detect the walking or movement. However, this method will not work properly on a person who is immobile and only breathing, such as the situation when a person is asleep. Another critical example is baby monitoring applications: inside the car (in-cabin) elimination of the forgotten baby syndrome, in-crib detection of sudden infant death syndrome (SIDS), both relies on accurate, true presence detection.

The topic of human presence detection using radars had been studied before both in the context of moving and stationary subjects. In [1], the authors showed the possibility of presence detection with a FMCW 24 GHz radar and compared it to passive-infrared (PIR) measurements. They utilized an energy detector on the range-Doppler map to decide whether a target is present or not, and did not deal with complete stationary or sleeping targets. The algorithm proposed in [2] is based on calculation of the Doppler power to identify doorway crossing and thus infer on the occupancy of a given room or space. This work also dealt with the problem of walking humans only. WiFi signals were used in [3] for passive-occupancy detection of humans. Though they did not deal with stationary targets, they reported promising results on people counting and presence detection of walking humans. Fourier processing with constant energy threshold detector was utilized in [4], however, stationary human presence accuracy was not reported.

The feasibility of using breathing to detect presence was proven in [5,6], where they showed that a mechanical target which simulates a breathing human can be detected in a room with 93% accuracy by using a Doppler radar with a threshold on the root mean square (RMS) of the received signal, while in [7] a non-adaptive energy threshold detector calculated from pre-recorded noise data was employed to determine if there is one person or two in the radar search volume, both in movement and stationary settings. However, high energy does not mean breathing, or presence. Finally, the usage of convolutional neural networks (CNN) were investigated in [8], where a Doppler radar and and infrared imaging device were jointly employed for presence detection of one human in a specific room. They reported an accuracy of 98.9%. The usage of CNNs bares a complex implementation for real-time purposes, as well as it relies heavily on visual information from the infrared imaging device.

Another application in which sensing the vital signs of a human subject is critical is through-wall and through-debris life sensing. In [9], the detection of vital signs through walls was investigated. They showed the feasibility of detecting the breathing and heart-rate of a human subject standing behind a wall, while in [10] they proposed a continuous wave radar architecture for the purpose of detecting vital signs through highly dense construction materials of about 1.5 m thick. In [11], they used empirical mode decomposition to prove feasible the detection of breathing, hand waving and body bending behind an obstacle emulating debris. Through-debris breathing detection was also shown feasible in [12], where they experiment with a debris setup and a live person lying and breathing under it. They showed visual results that the human can be detected, though they did not show how to detect the human in an automated manner. The underlying assumption of the above papers is that the subject is there. The accurate estimation of the breathing frequency is also of importance for many applications, including baby monitoring, elderly care, sleep monitoring and more.

Breathing rate extraction with a pulse-Doppler architecture was presented in [13], where they visually showed feasibility of extracting the breathing rate of a stationary and moving human using Fourier analysis. They employed range-Doppler processing, but there was no outline of how to detect the ’breathing targets’ and verify that it is in fact breathing, also the accuracy was not evaluated. The authors in [14,15] used the wavelet transform to overcome the discrete Fourier transform (DFT) resolution insufficiency, and for the same reason the chirp Z transform was used in [16] to estimate breathing rate. The chirp Z transform was also used in [17] coupled with an analytical model for the remote estimation of both breathing and heart-rate. The accuracy of these methods and a comparison against a known bound was not analyzed. More recent work on remote breathing extraction can be found in [18,19], in which breathing was extracted with a radar and a verification that the peak is falling within the breathing band of frequencies was done. While [18,19] reported accuracy results and evaluation of proposed methods, as we show in this paper, we achieve better results by using a maximum likelihood estimator. Furthermore, the verification presented there is not optimal, and does not test adherence to a breathing model, as the detector we present in this paper does.

The need to rely on the breathing movement for human presence detection stems from the fact that the use of a moving target indicator (MTI) [20], often fails to detect stationary humans. MTI is essentially a high-pass filter (HPF) that filters out close to zero-Doppler targets. This HPF caveat is that it most often filters out the breathing, being that breathing frequency is very close to the zero frequency (DC), hence, will fail to detect a sleeping person.

We propose an algorithm that detects presence using the minute movements of the abdomen and torso due to breathing, while at the same time, estimates the breathing frequency. Since detecting moving targets, even targets that slightly shift in their chair, can be done using MTI, we intentionally deal only with strictly stationary subjects.

Thus, this paper’s contribution is three fold. First, we present a framework of detecting presence using only the breathing movement. We develop a GLRT detector which, as an input, takes the suspected breathing pattern and as an output decides whether its breathing (presence) or not. Second, a maximum likelihood (ML) estimator of the breathing rate is developed, and shown to asymptotically achieve the Cramer–Rao lower bound (CRB), and lastly, we show that the GLRT detector and ML estimator are the same mathematical expression so we inherently get both with one evaluation. We tested this algorithmic framework performance on various scenarios such as sleeping babies and stationary adults, and results are reported.

In Section 2, we explain the measurement setup, and immediately move to derive both the GLRT detector as well as the ML estimator in Section 3. The estimator is also compared to the CRB. In Section 4, we explain the experiments we have done to verify our algorithms as well as report the results. We discuss future work in Section 5 and we conclude the paper in Section 6.

## 2. Measurement Setup

The radar we use is an ultra wide-band (UWB) radar module named X4M300 (Novelda AS, Oslo, Norway) which carries XeThru X4 UWB radar chip. We collect the raw data to the PC through USB. The data are then feeding the algorithm we propose. The radar parameters are depicted in Table 1.

The radar manufacturer tested this hardware for the specific application of breathing movement sensing for different angles and ranges. They concluded that even when the torso is 90 degrees rotated towards the radar, the micro-Doppler of the breathing is still present, and can be reliably detected even from 1.8 m away [21].

### Radar Operation

The radar is transmitting a pulse with a pulse repetition frequency of 40.5 MHz and receiving the returned pulse. Integration is done on many pulses in order to increase the signal to noise ratio (SNR) so that the output is an integrated pulse, called a frame, every 0.1 s. In general, the return signal represents a superposition of reflected pulses from the environment, including, if present, a breathing target. Each sample of this returned frame represents a range bin or a fast-time bin. Let $N_{rg}$ be the number of range bins. If we wait *T* seconds in slow time or *K* frames we will get a slow time vs. fast-time matrix of size $K\times N_{rg}$, in which each row is a radar frame and each column is the change of radar return amplitude over *T* seconds (and *K* radar frames) of slow time.

If we know the specific range bin in which the breathing phenomenon is present and extract this column, then we will get a slow time signal that is periodic with a fundamental breathing frequency $f_{b}$. The method to extract the relevant range-bin is detailed in the next section.

## 3. Method

### 3.1. Extracting the Suspected Breathing Signal

Let $D_{l}$ denote the matrix of size $K\times N_{rg}$ as described above, *l* is the current frame index such that the last row in $D_{l}$ is the *l*th received radar frame and the first row is the (l−K+1) frame.

The columns of this matrix are then filtered with a HPF that filters out all frequencies below a minimal breathing frequency of 0.15 Hz. Next, each column spectrum is calculated using the fast Fourier transform (FFT), generating a range-Doppler map.

A maximum peak is then searched for inside the range Doppler map, and its corresponding range-bin is declared as the range bin of the target. The respective column out of the matrix $D_{l}$ is extracted and is denoted in this paper as the signal x.

This signal x is then used in to estimate breathing frequency while at the same time to detect the breathing signal, as seen in the next subsections.

### 3.2. Fourier Series Expansion

In this section, we show how we represent the extracted signal using Fourier series (FS), which constructs the detection and estimation problem as a least squares problem. Since the extracted signal is suspected to be a periodic breathing signal with a deterministic period inside the observation window, we can represent it as a FS model

(1) $$x\left(k\right)=\sum_{m=-M}^{M}\theta_{m}\exp\left(-j2\pi mf_{b}k\right)+n\left(k\right),$$

where n(k) is a zero mean circularly complex Gaussian r.v. with variance $\sigma^{2}$, $f_{b}$ is the breathing frequency, $\theta_{m}$ is the *m*’th Fourier coefficient and *M* is the number of relevant breathing harmonies in the signal. Note that $f_{b}$ is unknown as well as the Fourier coefficients $\theta_{m}$. Arranging (1) in a vector-matrix form to account for k=0⋯K−1 samples we can write

(2) $$x=H\left(f_{b}\right)\theta +n,$$

where $x={\left[x(0),\cdots ,x(K-1)\right]}^{T}$, the k,m entry in the matrix $H\left(f_{b}\right)$ is given by ${\left[H\left(f_{b}\right)\right]}_{km}=\exp\left(-j2\pi mf_{b}k\right)$, $\theta ={\left(\theta_{0},\cdots ,\theta_{K-1}\right)}^{T}$ and $n={\left[n(0),\cdots ,n(K-1)\right]}^{T}$, thus, $n∼\mathrm{CN}\left(0,\sigma^{2}I\right)$.

### 3.3. Maximum Likelihood Estimation of the Breathing Rate

#### 3.3.1. Derivation of the ML Estimator

Define the parameters in (2) as $\vartheta ={\left(f_{b},\theta^{T}\right)}^{T}$, then the ML estimator of ϑ is given by

(3) $${\hat{\vartheta }}_{ML}=\arg\left\{\underset{f_{b},\theta }{min}{∥x-H(f_{b})\theta ∥}_{2}^{2}\right\}.$$

Note that the matrix $H(f_{b})$ is a function of the parameter $f_{b}$, thus, for the sake of brevity will be referred to as H in the sequel.

Since the ML estimator of the coefficient vector θ for a given $f_{b}$ is given by ${\hat{\theta }}_{ML}={\left(H^{H}H\right)}^{-1}H^{H}x$, inserting it back into (3) will yield

(4) $${\hat{f_{b}}}_{ML}=\arg\left\{\underset{f_{b}}{min}{∥x-H{\left(H^{H}H\right)}^{-1}H^{H}x∥}_{2}^{2}\right\}.$$

Since the term $H{\left(H^{H}H\right)}^{-1}H^{H}$ in (4) is the projection matrix into the column space of H, we define $P_{H}=H{\left(H^{H}H\right)}^{-1}H^{H}$. Moreover, since $I-P_{H}$ is also a projection matrix, it satisfies $\left(I-P_{H}\right)\left(I-P_{H}\right)={\left(I-P_{H}\right)}^{H}\left(I-P_{H}\right)=I-P_{H}$, we can write

(5) $${\hat{f_{b}}}_{ML}=\arg\left\{\underset{f_{b}}{min}{∥\left(I-P_{H}\right)x∥}_{2}^{2}\right\},$$

but

(6) $$\begin{array}{ccc} {∥\left(I-P_{H}\right)x∥}_{2}^{2} & = & x^{H}\left(I-P_{H}\right)x=\\ & = & x^{H}x-x^{H}P_{H}x=\\ & = & x^{H}x-{∥P_{H}x∥}_{2}^{2}, \end{array}$$

where the last line in (6) is due to the property that a projection matrix P will satisfy $P=P^{2}=P^{H}$.

Since the term $x^{H}x$ is independent of the parameter we can write

(7) $${\hat{f_{b}}}_{ML}=\arg\left\{\underset{f_{b}}{max}{∥P_{H}x∥}_{2}^{2}\right\},$$

which can be solved by a line search across valid values of the breathing rate $f_{b}\in \left[0.1,1.0\right]$ Hz.

We will see in the next subsection that the value of ${∥P_{H}x∥}_{2}^{2}$ evaluated at ${\hat{f_{b}}}_{ML}$ is also the GLRT value, so we get both the breathing rate estimation as well as the evaluation of the GLRT test value in one calculation.

#### 3.3.2. Cramér–Rao Lower Bound

The CRB for the estimation of the breathing frequency can be divided to different cases, dependent on which parameters are assumed to be known. In our case, we are only interested in the frequency $\omega_{b}=2\pi f_{b}$ while the amplitudes and phases are unknown and nuisance. For this case, the CRB for estimating $\omega_{b}$ is given by [19,22]

(8) $$\mathrm{CRB}\left({\hat{\omega }}_{b}\right)=\frac{12}{{\left({\mathrm{BW}}_{\mathrm{eff}}\right)}^{2}\times K^{3}\times \mathrm{SNR}},$$

where *K* is the number of samples, the SNR is defined in (9) and the effective bandwidth, $BW_{\mathrm{eff}}$ is defined in (10).

(9) $$\mathrm{SNR}\overset{def}{=}\frac{1}{2\sigma^{2}}\sum_{m=-M}^{M}\theta_{m}^{2},$$

(10) $${\mathrm{BW}}_{\mathrm{eff}}\overset{def}{=}\frac{1}{\sum_{m=-M}^{M}\theta_{m}^{2}}\sum_{m=-M}^{M}m^{2}\theta_{m}^{2}.$$

The effective bandwidth is also knows in the literature as r.m.s. bandwidth [23]. As seen in (8)–(10) if more harmonies are presented in the signal, the SNR effective bandwidth increases and the CRB decreases in an inverse relation.

**Figure 1:**
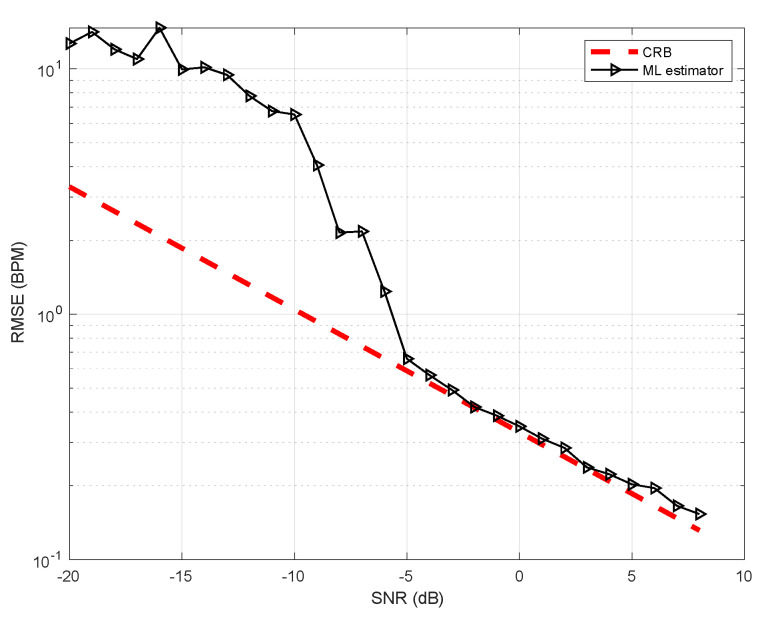
Root mean squared error vs. CRB for number of harmonics M=1 and fundamental frequency of 0.312 Hz.

The performance of the estimator in (7) for various SNR values compared to the CRB is depicted in Figure 1, where it is shown that for SNR>−5 dB the estimator meets the bound.

### 3.4. Generalized Likelihood Ratio Test (GLRT)

As derived in Appendix A the test is given by

(11) maxfbPHx22H1≷H0γ,

where γ is the test threshold and is computed in our experiments to a constant false alarm rate of $P_{FA}<10^{-7}$. The calculation of this threshold was done empirically and numerically using Neyman-Pearson’s theorem [24].

**Figure 2:**
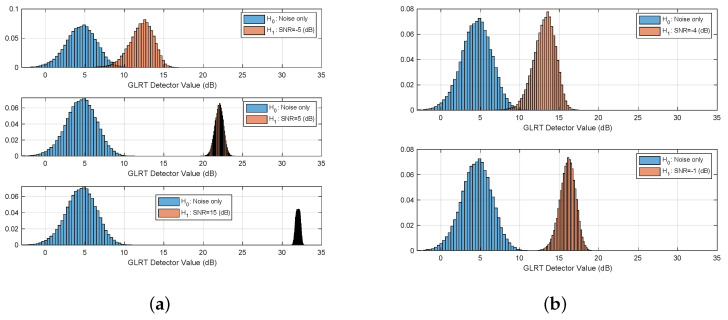
Histograms of the detector value for both hypotheses and various SNRs. (**a**) SNR =−5,+5,+15 dB. (**b**) SNR=−4,−1 dB.

**Figure 3:**
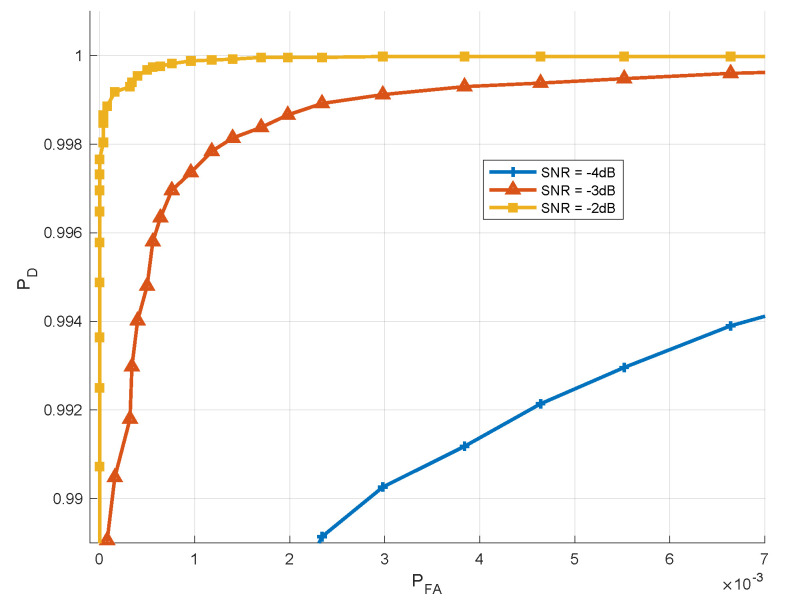
ROC Curve—Detection Probability vs. False alarm for various SNR values.

**Figure 4:**
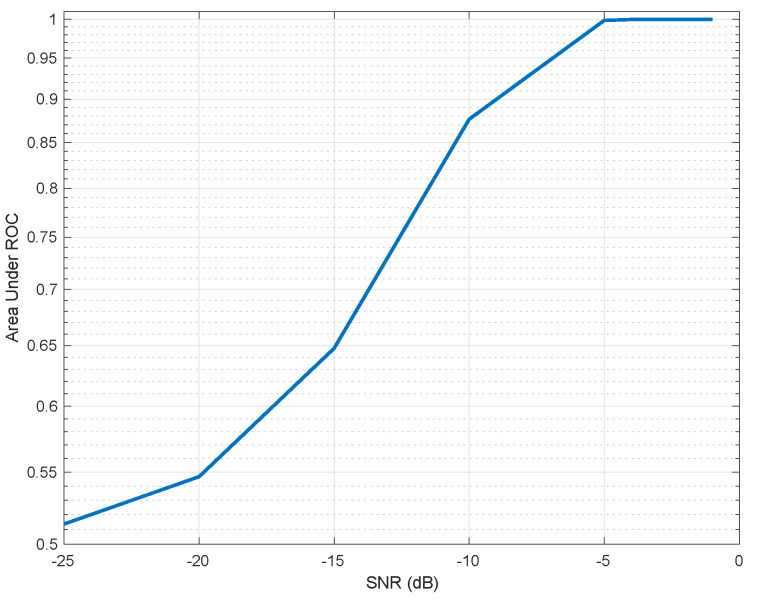
Area Under Curve for various SNR values.

Monte-Carlo simulation was performed in order to study the separation between hypotheses. Thus, histograms of the detector value, under both hypotheses for various SNRs is shown in Figure 2. As shown, a very good separation is achieved for SNR greater than −5 dB. The Receiver Operating Characteristics (ROC) curve of the test for various SNR values is given in Figure 3. As depicted for SNR =−5 dB we might be able to estimate the breathing frequency and meet the CRB but at the operating point we will have quite a few misses and false-alarms in detection. For lower SNR, the values of the area under the (ROC) curve or AUC is more informative. It can be seen that as expected for very low (∼−20 dB) SNR regime the AUC is about 0.5 meaning there is no separation capability between the hypotheses, but as we go up in SNR and close to −5 dB the near 1 AUC suggest a very good separation between the hypotheses as shown in Figure 4.

## 4. Experimental Results

**Figure 5:**
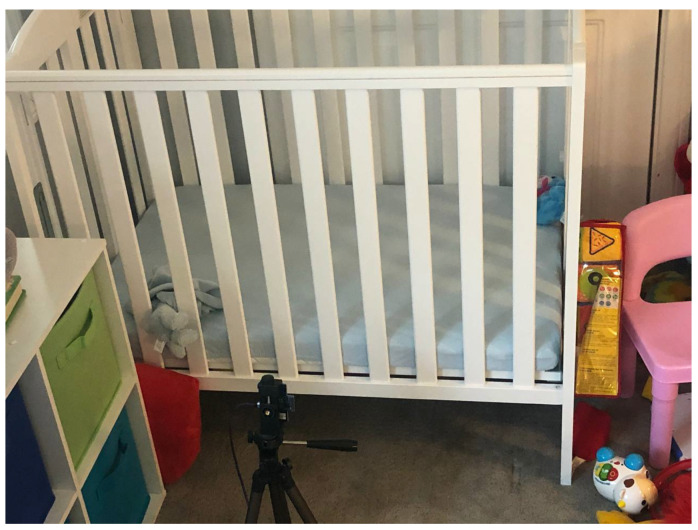
Baby crib—radar setup.

**Figure 6:**
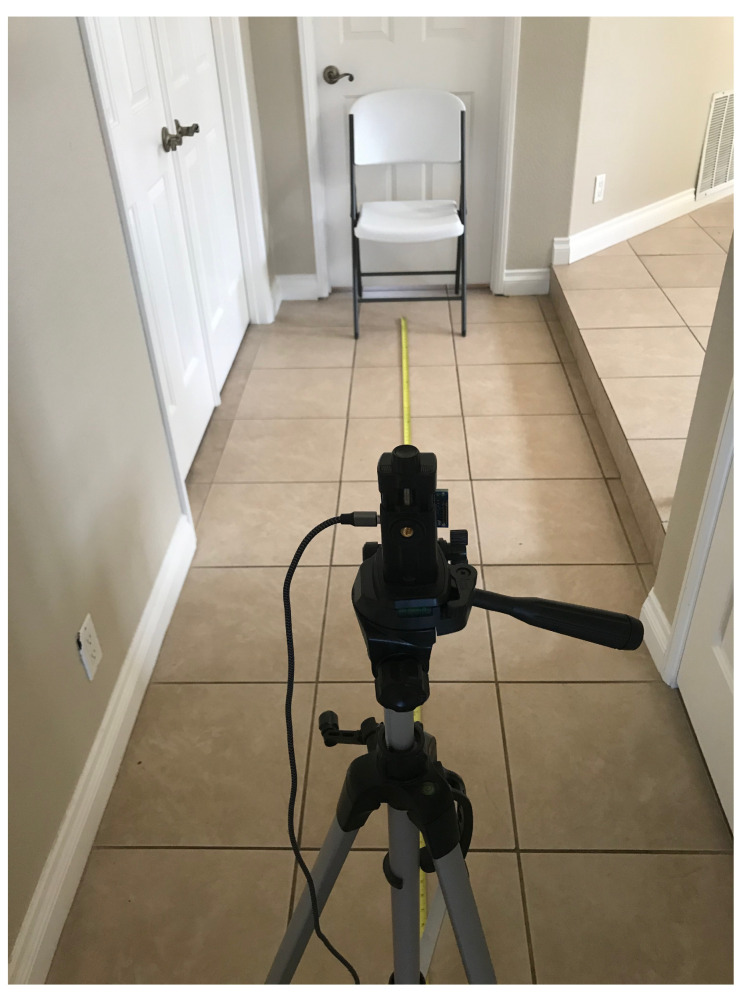
Breathing rate estimation—test setup.

The algorithm was tested in two distinct settings (Experiments approved by IRB number FES-HSES1901):

1. Baby sleeping. The proposed method was tested on 2 babies across 4 nights, each of which representing an uninterrupted, full night sleep, so total of 8 nights were tested. We placed the radar at half a meter and one meter away from the crib directly facing its long-side as depicted in Figure 5. Both babies were wearing a swaddle, and the babies were moving in the crib so random poses with respect to the radar were presented during the night. Analysis of the SNRs and test distribution are described below. No false alarms or misses were reported.
2. Adults sitting: The proposed method was tested on nine test subjects, ages 12 to 44, sitting still without moving in front of the sensor, in various distances, breathing. The duration was approx. 60 s and sometimes more per test subject. The purpose of this these experiments was to analyze the errors in breathing rate as reported below. The setup is as seen in Figure 6. The test subject was sitting on the chair, leaning back, sitting as still as they can, looking straight to the radar. The distance was measured both with the radar as well as a measuring tape as seen in the figure.

### 4.1. Efficacy of Breathing Signal Extraction

**Figure 7:**
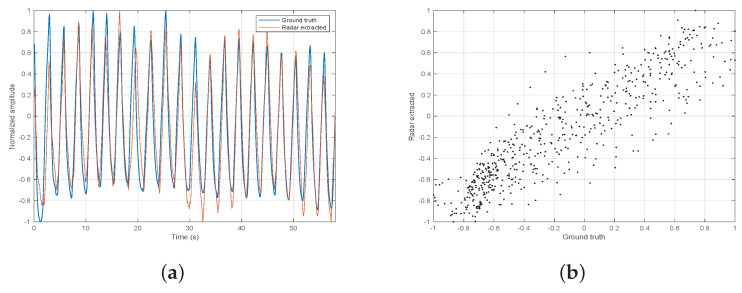
Experiment 1 signal extraction. (**a**) ground truth and radar extracted on top of each other. (**b**) correlation scatter.

**Figure 8:**
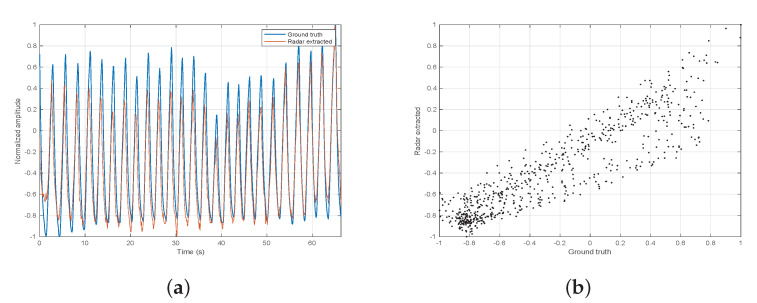
Experiment 2 signal extraction. (**a**) ground truth and radar extracted on top of each other. (**b**) correlation scatter.

**Figure 9:**
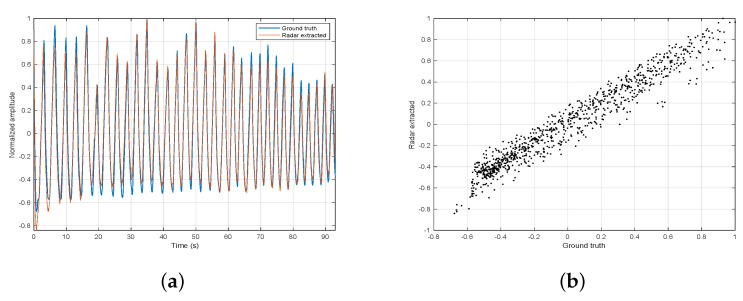
Experiment 3 signal extraction. (**a**) ground truth and radar extracted on top of each other. (**b**) correlation scatter.

The efficacy of the breathing signal extraction was discussed in [18]. It was tested on various subjects wearing on their abdomen a Neulog’s respiration monitoring Belt logger NUL-236 (Neulog, Rochester, NY, USA) [25], used as ground truth. In [18], the efficacy of the extracted breathing signal was not tested under various distances so we decided to further test it with different radar ranges, from 1 m to 4 m. Table 2 summarizes the subjects parameters, while Table 3 shows the correlation coefficients of the radar extracted breathing signal to the ground truth. A few examples of the two signals superimposed on each other are shown in Figure 7, Figure 8 and Figure 9. We report a maximum correlation of 0.971 and minimum of 0.781.The mean correlation coefficient we get over all distances and subjects is 0.881 which suggests a high efficacy of breathing signal extraction.

### 4.2. Examples of the Test Distribution for Various SNR Regimes

**Figure 10:**
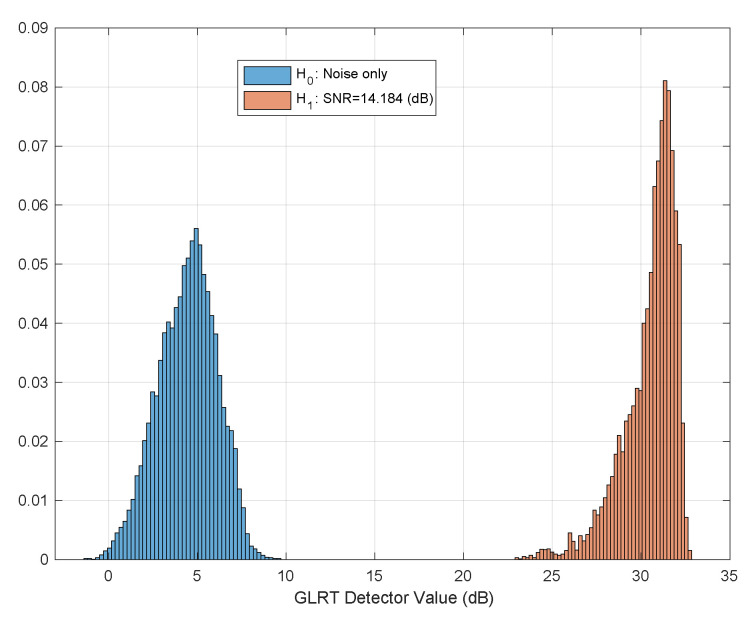
Real example of test hypothesis separation. SNR ∼=14 dB. Extracted from an overnight sleep of Baby I.

**Figure 11:**
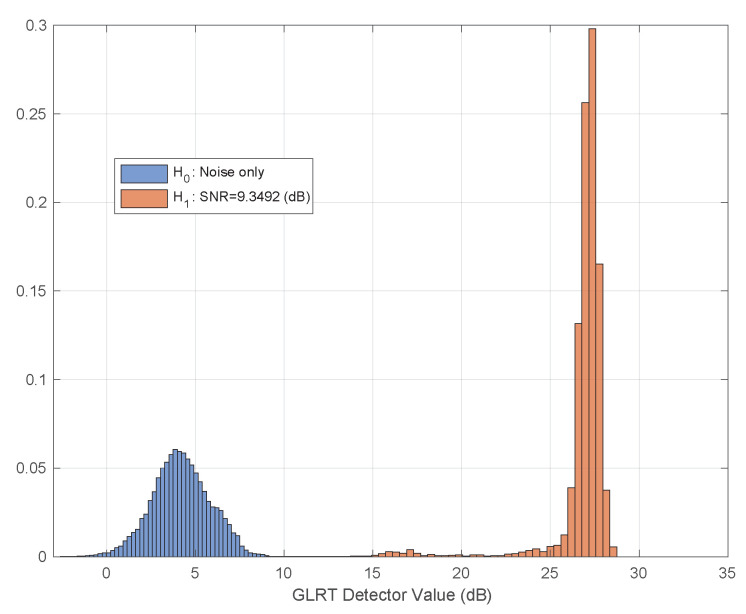
Real example of test hypothesis separation. SNR ∼=9 dB. Extracted from an overnight sleep of Baby II.

Baby I and II (see Table 4) were recorded sleeping for 4 nights each. We placed the radar at distances of half a meter and a meter away from the crib directly facing its long-side as depicted in Figure 5. The two different scenarios make for a variation in estimated SNR which we can use to visually test the adherence of the test distribution under both hypotheses to the theoretical above. As can be seen in Figure 10 and Figure 11 the graphs resembles the theoretical results depicted in Figure 2 for SNR =9,14 dB.

### 4.3. Accuracy Results

This section will outline the detector performance and ML estimation accuracy results. In our experiments we took M=2 harmonies and a time window of 10 s or K=100 samples. The time window affects the estimation accuracy as per Equation (8). Shortening it will provide less information in the observation window and the estimation accuracy will drop, increasing it will improve it but at the same time increase the risk of losing local stationarity, i.e., breathing rate change inside the time window. Ten seconds is what we chose as a trade-off.

#### 4.3.1. Detector Accuracy

The detector accuracy was evaluated across 8 combined nights of sleeping, split between two baby subjects. The ground truth was a camera synced to the radar. Over these 8 nights no false alarm or misses were reported. The reason for that is we work on a good SNR regime in which the separation between hypotheses is good and the sensitive detector probably needs months of operations to have a false or a miss. Figure 10 and Figure 11 depicts the real, empirical, distribution of the test under both hypotheses. The babies move and change pose throughout the night, thus, the test value has a range of between 14 dB to 33 dB.

#### 4.3.2. ML Estimation Accuracy

The results of the experiments described above performed on the nine subjects in Table 2 are shown in Table 5, Table 6 and Table 7. The root mean squared error (RMSE) in BPM for different distances are summarized. At times, the subjects were instructed to either breathe normally, or fast, to allow for variations in breathing rate. The table layout the breathing rate, the RMSE in BPM and the percentage of the RMSE with respect to the true breathing rate. The true breathing rate is reported and any rate higher than 20 BPM is considered fast. We report maximal error of 0.170 BPM and maximum percentage of 0.968% of true breathing rate across all experiments and subjects, which is much better than the performance of the algorithm we proposed in [18].

## 5. Future Work

The below points were identified for future work.

### 5.1. Sleep Stages Classification

The proposed algorithm is dealing only with presence detection, hence, there is a spread in values of the detector across time as shown in Figure 10 and Figure 11. It is interesting to investigate if the GLRT value along with the breathing rate can be used as a descriptor to classify sleep stages, like awake, deep, light sleep, and Rapid Eye Movement sleep (REM).

### 5.2. Breathing Extraction under Movements

The proposed algorithm is detecting the movement of the breathing, however, when shifting in the chair or in bed, the breathing is “masked” by the bulk movement of the body. While a simple MTI will detect the movement, the breathing estimation during this movement will be wrong, because the movement is not taken into account in the proposed breathing model. Investigation of how to deal with remote breathing extraction under movement is something we plan for the future.

## 6. Conclusions

A method for the simultaneous presence detection and breathing rate estimation was presented. The method relies on the detection of minute movements of the torso due to breathing, such that even when the person is sitting still and breathing only, the detector is still effectively able to tell that there is a presence. The algorithm expands the breathing signal using Fourier series, and uses a simple line search for the maximum likelihood estimation of the breathing rate and the detector value simultaneously. We analyzed both the detector’s performance as well as the estimator’s performance, both in a Monte-Carlo setting as well as real life, and concluded that the estimator meets the CRB and for SNR values greater than −5 dB. Moreover, we report a maximum error of 0.170 BPM for distances up to 4 m. In order to analyze the detector performance in real life, we tested the algorithm on eight full nights recordings of two babies sleeping, reporting zero false alarm and zero misses.

## Figures and Tables

**Table 1:** Radar parameters.

	Value	Units	Comment
**Pulse Repetition Frequency**	40.5	MHz	
**Center frequency**	7.29	GHz	
**Bandwidth**	1.5	GHz	10 cm range resolution
**Peak pulse power**	−0.7	dBm	
**Azimuth field of view**	120	Degrees	
**Elevation field of view**	115	Degrees	
**Frame rate**	∼10	Hz	∼70 dB processing gain

**Table 2:** Subjects information.

	Gender (m/f)	Age (years)	Weight (kg)	Height (cm)
Subject 1	m	44	96	174
Subject 2	f	40	63	173
Subject 3	f	12	35	145
Subject 4	f	25	60	170
Subject 5	f	28	57	166
Subject 6	f	31	56	162
Subject 7	m	35	77	178
Subject 8	m	41	83	181
Subject 9	m	43	169	175

**Table 3:** Experiment scenarios correlation coefficients.

Experiment Number	Subject	Distance (m)	Correlation Coefficient	True Breathing Freq (BPM)
1	1	1.13	0.905	24.023
2	1	1.83	0.971	19.921
3	1	2.56	0.929	20.507
4	2	1.54	0.848	16.406
5	2	3.54	0.815	15.234
6	3	1.21	0.864	25.19
7	3	2.68	0.940	25.78
8	3	4.046	0.781	31.640

**Table 4:** Baby information.

	Gender (m/f)	Age (months)	Weight (Kg)	Approx Radar Range (m)
**Baby I**	m	10	11.2	0.5, 1.0
**Baby II**	f	19	11.5	0.5, 1.0

**Table 5:** Root mean squared error results on test subjects for ranges 1–2 m.

Subject	Distance (m)	RMSE (BPM)	True Breathing Freq	Mean RMSE % of Breathing Freq
**1**	1.13	0.095	21.093	0.450
**1**	1.83	0.069	19.921	0.346
**2**	1.54	0.131	16.406	0.798
**3**	1.21	0.100	25.190	0.396
**4**	1.32	0.110	14.010	0.785
**5**	1.61	0.129	13.121	0.983
**6**	1.51	0.125	16.643	0.751
**7**	1.42	0.099	16.432	0.602
**8**	1.70	0.131	15.327	0.854
**9**	1.30	0.119	17.282	0.688

**Table 6:** Root mean squared error results on test subjects for ranges 2–3 m.

Subject	Distance (m)	RMSE (BPM)	True Breathing Freq	Mean RMSE % of Breathing Freq
**1**	2.56	0.096	20.507	0.468
**2**	2.47	0.151	18.981	0.795
**3**	2.68	0.100	25.780	0.387
**4**	2.31	0.120	15.001	0.799
**5**	2.76	0.099	14.132	0.700
**6**	2.81	0.133	16.320	0.814
**7**	2.90	0.148	18.982	0.779
**8**	2.66	0.129	15.903	0.811
**9**	2.24	0.090	16.837	0.534

**Table 7:** Root mean squared error results on test subjects for ranges 3–4 m.

Subject	Distance (m)	RMSE (BPM)	True Breathing Freq	Mean RMSE % of Breathing Freq
**1**	3.19	0.139	20.732	0.670
**2**	3.54	0.146	15.236	0.958
**3**	4.04	0.170	31.640	0.537
**4**	3.85	0.138	14.390	0.958
**5**	3.96	0.130	13.417	0.968
**6**	3.78	0.115	15.901	0.723
**7**	3.07	0.129	17.003	0.758
**8**	3.60	0.141	15.390	0.916
**9**	3.66	0.135	17.100	0.789

## Appendix A. Derivation of the GLRT Detector

The hypotheses testing problem we are dealing with here can be written as

(A1) $$\begin{array}{ccc} H_{1} & : & x=H\theta +n\\ H_{0} & : & x=n, \end{array}$$

where H of size N×N and θ of size N×1 are unknown deterministic parameters and n is a vector of noise such that in the general case $n∼\mathrm{CN}\left(0,R_{n}\right)$.

Using maximum likelihood estimation of the parameters, the GLRT is given by

(A2) maxfb,θfx;H,θfx;Hθ=0H1≷H0γ1.

If we substitute the ML estimator of H and θ we get

(A3) fx;H^,θ^fx;Hθ=0H1≷H0γ1.

The distribution of x under each hypothesis is complex Gaussian and is given by

(A4) $$\begin{array}{ccc} x/H_{1} & & ∼\mathrm{CN}\left(\hat{H}\hat{\theta },R_{n}\right),\\ x/H_{0} & & ∼\mathrm{CN}\left(0,R_{n}\right). \end{array}$$

The Gaussian under both hypotheses has the same multiplicative coefficient, hence after taking the natural logarithm of (A2), omitting the max operation for brevity, we get

(A5) −x−Hθ^HRn−1x−Hθ^+xHRn−1xH1≷H0γ2.

Since the ML estimator of θ, denoted here by $\hat{\theta }$, is given by the weighted least squares estimator we can write

(A6) $$\hat{\theta }={\left(H^{H}R_{n}^{-1}H\right)}^{-1}H^{H}R_{n}^{-1}x,$$

and

(A7) $$\begin{array}{ccc} & x & -H\hat{\theta }=\\ & = & x-H{\left(H^{H}R_{n}^{-1}H\right)}^{-1}H^{H}R_{n}^{-1}x=\\ & = & R_{n}^{\frac{1}{2}}\left[R_{n}^{-\frac{1}{2}}x-R_{n}^{-\frac{1}{2}}H{\left(H^{H}R_{n}^{-1}H\right)}^{-1}H^{H}R_{n}^{-\frac{1}{2}}R_{n}^{-\frac{1}{2}}x\right]=\\ & = & R_{n}^{\frac{1}{2}}\left[\left(I-R_{n}^{-\frac{1}{2}}H{\left(H^{H}R_{n}^{-1}H\right)}^{-1}H^{H}R_{n}^{-\frac{1}{2}}\right)R_{n}^{-\frac{1}{2}}\right]x. \end{array}$$

Since $P_{\tilde{H}}=R_{n}^{-\frac{1}{2}}H{\left(H^{H}R_{n}^{-1}H\right)}^{-1}H^{H}R_{n}^{-\frac{1}{2}}$ is a projection matrix into the column space of $\tilde{H}=R_{n}^{-\frac{1}{2}}H$, and $P_{\tilde{H}}^{⊥}=I-P_{\tilde{H}}$ is also a projection matrix, hence, noting that any projection matrix P will satisfy $P=P^{2}=P^{H}$ we can write

(A8) $$\begin{array}{c} x-H\hat{\theta }=R_{n}^{\frac{1}{2}}\left(I-P_{\tilde{H}}\right)R_{n}^{-\frac{1}{2}}x, \end{array}$$

and

(A9) $${\left(x-H\hat{\theta }\right)}^{H}=x^{H}R_{n}^{-\frac{1}{2}}\left(I-P_{\tilde{H}}\right)R_{n}^{\frac{1}{2}}.$$

Thus, calculating the first term in the left hand side (LHS) of (A5) we get

(A10) $$\begin{array}{ccc} & - & {\left(x-H\hat{\theta }\right)}^{H}R_{n}^{-1}\left(x-H\hat{\theta }\right)=\\ & = & -x^{H}R_{n}^{-\frac{1}{2}}\left(I-P_{\tilde{H}}\right)R_{n}^{\frac{1}{2}}R_{n}^{-1}R_{n}^{\frac{1}{2}}\left(I-P_{\tilde{H}}\right)R_{n}^{-\frac{1}{2}}x=\\ & = & -x^{H}R_{n}^{-\frac{1}{2}}\left(I-P_{\tilde{H}}\right)R_{n}^{-\frac{1}{2}}x. \end{array}$$

Plugging this result into the LHS of (A5) we get

(A11) $$\begin{array}{ccc} & - & x^{H}R_{n}^{-\frac{1}{2}}\left(I-P_{\tilde{H}}\right)R_{n}^{-\frac{1}{2}}x+xR_{n}^{-1}x=\\ & = & x^{H}R_{n}^{-\frac{1}{2}}P_{\tilde{H}}R_{n}^{-\frac{1}{2}}x={∥P_{\tilde{H}}R_{n}^{-\frac{1}{2}}x∥}_{2}^{2}. \end{array}$$

Setting $R_{n}=\sigma^{2}I$, re-instating omitted the $max\left\{\cdot \right\}$ operation and moving the constant noise variance $\sigma^{2}$ into the RHS to be absorbed in the new threshold γ brings us to the next expression which concludes our derivation.

(A12) maxfbPHx22H1≷H0γ.
